# A Long Journey before Cycling: Regulation of Quiescence Exit in Adult Muscle Satellite Cells

**DOI:** 10.3390/ijms23031748

**Published:** 2022-02-03

**Authors:** Shaopu Zhou, Lifang Han, Zhenguo Wu

**Affiliations:** 1 State Key Laboratory of Molecular Neuroscience, Division of Life Science, Hong Kong University of Science and Technology, Clearwater Bay, Kowloon, Hong Kong, China; szhouam@connect.ust.hk (S.Z.); lhanac@connect.ust.hk (L.H.); 2 Greater Bay Biomedical Innocenter, Shenzhen Bay Laboratory, Shenzhen 518055, China

**Keywords:** satellite cells, quiescence exit, cell growth, checkpoints, mTORC1, cell cycle re-entry

## Abstract

Skeletal muscle harbors a pool of stem cells called muscle satellite cells (MuSCs) that are mainly responsible for its robust regenerative capacities. Adult satellite cells are mitotically quiescent in uninjured muscles under homeostasis, but they exit quiescence upon injury to re-enter the cell cycle to proliferate. While most of the expanded satellites cells differentiate and fuse to form new myofibers, some undergo self-renewal to replenish the stem cell pool. Specifically, quiescence exit describes the initial transition of MuSCs from quiescence to the first cell cycle, which takes much longer than the time required for subsequent cell cycles and involves drastic changes in cell size, epigenetic and transcriptomic profiles, and metabolic status. It is, therefore, an essential period indispensable for the success of muscle regeneration. Diverse mechanisms exist in MuSCs to regulate quiescence exit. In this review, we summarize key events that occur during quiescence exit in MuSCs and discuss the molecular regulation of this process with an emphasis on multiple levels of intrinsic regulatory mechanisms. A comprehensive understanding of how quiescence exit is regulated will facilitate satellite cell-based muscle regenerative therapies and advance their applications in various disease and aging conditions.

## 1. Overview

Skeletal muscle, comprising up to 40% of body weight of adult humans, maintains essential body functions including force generation, locomotion, posture maintenance, breathing, nutrient storage, metabolic regulation, and heat production. Skeletal muscle is composed of bundles of multinucleated myofibers formed by the fusion of mononucleated myoblasts. As a stable tissue under homeostasis, skeletal muscle possesses robust regeneration abilities in response to injury, a process relying on the muscle-resident adult muscle satellite cells (MuSCs) [1,2,3,4,5]. Adult myogenesis post injury is one of the most well-established paradigms to study the functions of stem cells and their contribution to tissue regeneration. MuSCs are sandwiched between basal lamina and plasma membrane of myofibers, and are so named due to their close proximity to myofibers. Since their discovery by Alexander Mauro in 1961 using electron microscopy [6], a plethora of molecular markers have been identified for MuSCs. PAX7, a paired domain and homeodomain-containing transcription factor, is one of the most widely-used MuSC markers [5,7,8,9]. Other commonly used markers for MuSCs include myogenic regulatory factors 5 (MYF5), as well as several cell surface proteins (e.g., CD34, α7-integrin, VCAM1, Calcitonin receptor (CALCR), c-Met, Syndecan4, etc.). Through transplantation and genetic ablation experiments, it is well established that satellite cells are bona fide stem cells indispensable for muscle regeneration [10,11,12,13,14]. Typically present in a quiescent G0 state in uninjured muscles under homeostasis, MuSCs are quickly activated and re-enter the cell cycle in response to acute muscle damage. After limited rounds of expansion, the majority of MuSCs differentiate by exiting the cell cycle and fuse with each other or existing myofibers. The MuSC pool is replenished by the self-renewal process through both asymmetric and symmetric cell divisions [5,15,16]. The regenerative capacities of MuSCs decline with aging, and their functions are impaired in genetically inherited muscle diseases such as the fatal Duchenne muscular dystrophy [17,18,19]. Interestingly, denervation-induced muscle atrophy also leads to MuSC activation and cell cycle re-entry [20,21]. However, the underlying mechanism remains less clear. On the other hand, MuSCs also actively contribute to the regeneration of neuromuscular junctions in response to denervation [22,23,24]. Therefore, a better understanding of the mechanisms underlying muscle regeneration and MuSC functions is warranted to develop muscle stem cell-based regenerative therapies for both aging and disease conditions. In this review, we discuss mechanisms governing MuSC quiescence and activation, with an emphasis on how the transition from quiescence into the first cell cycle is regulated.

## 2. Quiescence and MuSC Functions

Like hematopoietic stem cells (HSCs), hair follicle stem cells (HFSCs), or neural stem cells (NSCs), adult MuSCs are also known to exist in the quiescent state under homeostasis [2,4,25,26,27,28,29,30]. Proper regulation of quiescence is essential to maintain the functional stem cell reservoir and tissue regeneration capacity. Mutations that disrupt stem cell quiescence lead to loss of the stem cell pool and tissue regeneration capacities [31,32,33,34]. Quiescence represents a unique cellular state with reversible cell cycle arrest that is distinct from terminal differentiation or senescence, the cellular states with permanent cell cycle arrest [4,35,36]. Previously considered as a default cellular state, it has now been well accepted that quiescence is under active regulation by an intricate network that maintains stem cells in a poised state capable of rapid activation in response to stimuli. Advances in the techniques such as prospective stem cell isolation and high throughput transcriptomic profiling led to the identification of a molecular signature of quiescent MuSCs, which includes low expression of cell cycle related genes and high expression of niche remodeling genes [37,38]. While many features of the quiescent MuSC signature are shared among other quiescent stem cells such as HSCs and HFSCs, different types of stem cells do express unique molecular profiles and a common stemness signature for all adult stem cells does not seem to exist [36,39]. Much of our knowledge on cellular quiescence comes from studies in yeast and mammalian cell lines, where common strategies to induce cellular quiescence include nutrient and mitogen deprivation, loss of adherence as well as contact inhibition [4]. In such cells, quiescence is recognized as a cellular protection mechanism to prolong cell survival in response to a variety of stresses that negatively affect cell growth. Similarly, quiescence is adopted by adult stem cells as a protective mechanism to minimize cellular stresses including reactive oxygen species (ROS) and DNA damage, thereby enabling their long-term maintenance [40,41,42]. For example, quiescent MuSCs showed prolonged survival in adverse environment such as post-mortem tissues without losing stem cell functions [2,43]. Similar to their roles in cultured cell lines, many cell cycle regulators also critically regulate quiescence of stem cells. For example, RB is required to limit the activity of E2F transcription factors and cell cycle progression [44]. MuSC-specific loss of *Rb1* led to their uncontrolled expansion [45]. In HSCs, inactivation of individual RB family members failed to break quiescence, but deletion of all 3 RB family members is required to break HSC quiescence [46]. CDKN1A and CDKN1B are intrinsic inhibitors of cyclin dependent kinases (CDKs). In MuSCs, CDKN1B is required to preserve the more dormant label-retaining MuSC pool, while CDKN1A plays more important roles in non-label-retaining MuSCs [47]. CDKN2A, on the other hand, is only expressed in geriatric MuSCs and serves as a molecular switch controlling entry into senescence from a reversible quiescent state [18].

Apart from existing in a reversible G0 state, quiescent cells also exhibit several general features that distinguish them from cycling cells. Quiescent cells are small in size, accompanied with reduced levels of transcription and translation [4]. Metabolically, quiescent cells favor catabolism, such as autophagy and fatty acid oxidation (FAO), over anabolism [1,2,4,48]. Low levels of oxidative phosphorylation (OXPHOS) in mitochondria contribute to the fitness of quiescent cells by limiting the production of ROS and subsequent cellular damage. Autophagy also plays an important role in restraining ROS production by clearance of unhealthy or defective mitochondria. Defective autophagy induces an increase in ROS levels in MuSCs from geriatric mice, which triggers cellular senescence and loss of regenerative functions [49].

## 3. MuSC Heterogeneity

Although similarly marked by PAX7 expression, MuSCs from different muscle tissues have been shown to possess varied regenerative abilities. MuSCs from extraocular muscles (EOMs) have superior engraftment potentials compared to limb derived MuSCs [50]. Furthermore, MuSCs from the same muscle tissue also display transcriptomic and functional variations, suggesting that adult MuSCs represent a mixed pool of stem cells with varied features.

Through label retention experiments using H2B-GFP or BrdU, it has been found that quiescent MuSCs are a heterogenous population with different label-retaining properties and stemness, which could result from crosstalk between MuSCs and their local niches [47,51,52]. The heterogeneity occurs at different levels, including transcription factor/surface protein expression, metabolic properties, activation kinetics and stress resistance. In 2007, a study from Rudnicki group demonstrated that around 90% of adult MuSCs express *Myf5* and are committed to myogenic differentiation, whereas only 10% of the adult MuSC pool are satellite stem cells as they have never expressed *Myf5* and extensively contribute to the stem cell pool after transplantation [5,15]. Satellite stem cells can generate both satellite stem cells and *Myf5*^+^ satellite myogenic cells through apical-basal oriented asymmetric cell division. Interestingly, results from intravital imaging suggest that such apical-basal divisions might be rare during muscle regeneration as most cell divisions happen along the longitudinal axis of extracellular matrix remnants [53]. While all adult MuSCs are PAX7 positive, the expression levels of *Pax7* vary among MuSCs. Higher expression of *Pax7* was observed in a more dormant subgroup of MuSCs, which display reduced metabolic activities and delayed cell cycle re-entry compared to their *Pax7*^low^ counterparts [16]. Again, asymmetric cell division may play an essential role in establishing this cellular hierarchy. Interestingly, this *Pax7*^high^ population resembles the recently identified genuine quiescent MuSC state in terms of low metabolic activity and slow activation kinetics, which is maintained by high FoxOs activity and marked by higher CD34 expression [54]. Moreover, the expression of PAX3 also defines different subsets of MuSC population. PAX3^+^ MuSCs are endowed with higher resistance and survival advantages compared to PAX3^−^ populations in response to environmental stresses like toxic pollutant or irradiation [40,41]. Although PAX3 confers satellite cells with higher stemness resembling that of reserve or dormant stem cells, it probably marks a different population from *Pax7*^high^ or genuine quiescent MuSCs. A recent study from Rando group demonstrated that PAX3 expression is positively correlated with the activation rate of MuSCs, which is contrary to the slow activation feature of genuine quiescent MuSCs [16,54,55]. Apart from transcription factors, MuSC heterogeneity is also reflected by differential surface marker expression, while CD34 marks a more quiescent state of MuSCs, CD9 and CD44 are highly expressed in activated progenitor cells [56]. Similarly, Syndecan proteins, which are upregulated in cycling cells, are also heterogeneously expressed under quiescence, implicating the existence of different MuSC pools [57]. Human MuSCs are also heterogeneous in terms of CAV1 expression, and CAV1^+^ human MuSCs have enhanced engraftment ability compared to CAV1^-^ MuSCs [58].

Although previous studies have provided useful insights into functional diversities among MuSCs, the relationships among different MuSC populations remain elusive. The rapidly developing single cell techniques have opened a new avenue to systematically characterize the heterogeneities among MuSCs [57,59,60,61]. Two major MuSC subgroups have been identified by single cell RNA-seq (scRNA-seq). MuSCs close to quiescence (cQ) express higher levels of *Pax7* together with genes related to cell cycle arrest and stress resistance, while MuSCs prone to early activation (eA) express gene signatures involved in the ribosome biogenesis and mRNA processing [60]. Conclusions from this study are consistent with previous reports describing the existence of a more dormant MuSC sub-population within the entire MuSC pool [16,54]. Future multimodal single cell analyses integrating surface protein expression, chromatin accessibility, gene expression together with spatial information of cellular organization, will provide a more comprehensive understanding of how the heterogeneity is established among MuSCs.

## 4. Regulatory Mechanisms Underlying MuSC Quiescence Maintenance and Exit

**Figure 1:**
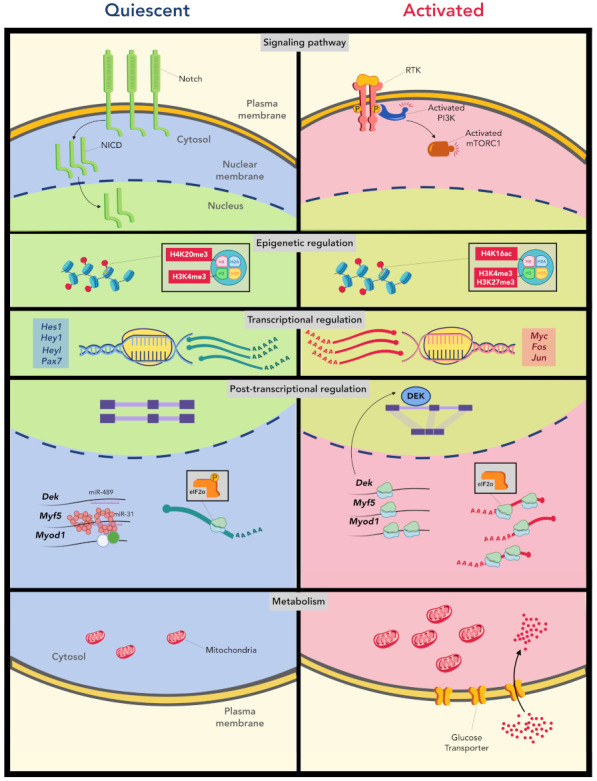
Regulatory mechanisms controlling MuSC quiescence maintenance and quiescence exit (activation). The balance between MuSC quiescence and activation is actively regulated via multiple levels of mechanisms. These mechanisms function at signaling, epigenetic, transcriptional, post-transcriptional and metabolic levels, and together determine the fate of MuSCs. Notch signaling is highly active under quiescence, which is replaced by PI3K signaling upon activation. The epigenetic landscape also differs significantly between quiescent and activated MuSCs. The metabolic reprogramming upon activation is orchestrated with utilization of distinct cellular metabolic pathways. In terms of transcriptional landscape, MuSC activation is accompanied with a drastic reduction of Notch targets and induction of many immediate early genes (IEGs). The post-transcriptional mechanisms, including intron retention and the microRNA pathways, are also essential to maintain MuSC quiescence while preserving their rapid response to injury cues.

An interesting question about MuSC heterogeneity is whether such heterogeneity reflects different depths of quiescence among MuSCs. It has been reported that MuSC quiescence comprises at least two different states: a dormant G0 state and a more primed G_alert_ state, and MuSC can reversibly transit between these two states through modulation of the mechanistic target of rapamycin (mTOR) signaling [62,63]. Different depths of cellular quiescence have been described in HSCs and NSCs as well. For example, the level of CDK6 in HSCs determines the difference between LT-HSCs and ST-HSCs. While both LT-HSCs and ST-HSCs are equally quiescent, a higher expression of CDK6 in ST-HSCs enables more rapid cell cycle entry [64]. scRNA-seq also revealed NSCs exist in a continuum of dormant and partially activated states [65]. The dormant NSCs (qNSC1) first enter the primed-quiescent state before becoming fully activated. These studies reinforce the concept that quiescence is not a homogeneous cellular state but covers a spectrum with different depths of quiescence. A comprehensive understanding of the underlying mechanisms will help develop and advance stem cell-based therapies for tissue regeneration. Here, we discuss recent progress in this topic and highlight regulatory mechanisms that function at different levels (Figure 1).

### 4.1. Signaling Pathways

Studies with MuSCs have revealed diverse signaling mechanisms working at different levels to regulate quiescence maintenance and exit (i.e., SC activation) [36,48]. Multiple signaling pathways have been found to regulate MuSC quiescence. Notch signaling is highly active in quiescent MuSCs but its activity drops precipitously upon SC activation [38,66], suggesting Notch activity is essential for the maintenance of quiescent MuSCs (Figure 1). Indeed, inducible deletion of *Rbpj* leads to spontaneous MuSC activation and differentiation at the expense of the quiescent MuSC pool [67,68]. Consistently, MuSCs deficient in both *Hey1* and *Heyl* (2 downstream targets of Notch signaling) display strong defects in quiescence maintenance [69]. In contrast, constitutive activation of Notch signaling via MuSC-specific overexpression of Notch intracellular domain (NICD) favors self-renewal of MuSC at the expense of subsequent SC activation, myoblast proliferation and differentiation [70]. One possible mechanism by which Notch maintains MuSC quiescence is its inhibition of myogenic differentiation: the HEY family transcription repressors are suggested to repress *Myog* gene expression [71]. Notch signaling also plays similar roles during embryonic myogenesis, where the precocious differentiation phenotype of the Notch deficient mutants could be rescued through co-deletion of *Myod1* [72,73,74]. Notch was also reported to contribute to quiescence by direct induction of *Pax7* transcription, a process independent of MyoD [70]. Other mechanisms by which Notch contributes to MuSC quiescence include activation of CALCR and suppression of cell migration, through induction of ColV and miR-708 expression, respectively [75,76]. The Notch signaling pathway is engaged by cell-to-cell contact as both the Notch ligands and receptors are transmembrane proteins. Therefore, the requirement for Notch signaling to maintain MuSC quiescence reinforces the importance of niche in preserving stem cell pools. Consistent with their close proximity to MuSCs, both mature myofibers and endothelial cells have been suggested to provide Notch ligands for MuSCs [77,78]. In contrast to Notch signaling, PI3K-AKT-mTORC1 signaling is inactive under quiescence but rapidly induced upon activation [62,79]. As discussed below, PI3K signaling is both necessary and sufficient to trigger the activation of quiescent MuSCs [33,79]. mTORC1 is an important downstream effector of PI3K signaling. It is a conserved Ser/Thr kinase belonging to the PI3K-related protein kinase family and serves as the catalytic core of two related complexes: mTORC1 and mTORC2 [80]. mTORC1 senses cellular energetic and nutritional status and directs cells into growth and proliferation under optimal conditions. mTORC1 directly stimulates multiple anabolic pathways including protein synthesis, lipid synthesis, nucleotide synthesis and simultaneously suppresses catabolic metabolisms such as autophagy [81]. On the other hand, mTORC2 controls processes like cytoskeleton re-organization and cell survival [81]. Full activation of mTORC1 requires its lysosomal localization, where it is activated by the GTP-bound GTPase RHEB [82]. Multiple environmental cues including growth factors and energy availability signal through TSC1/2, a GTPase-activating proteins (GAP) complex for RHEB. TSC1/2 is inhibited upon growth factor stimulation, which enables activation of mTORC1 by GTP-bound RHEB [83,84,85,86,87,88,89]. The lysosomal localization of mTORC1 is controlled by the availability of amino acids, among which Leu and Arg are particularly potent [90]. This process is mediated by 4 small GTPases, RagA, RagB, RagC and RagD. RagA and RagB are homologous and form a heterodimer with RagC or RagD. The GTP-bound RagA/B assembles with GDP-bound RagC/D in the presence of sufficient amino acids, directing mTORC1 to the surface of lysosome through its interaction with RAPTOR [91,92,93,94]. mTORC1 mediates the effect of PI3K in MuSCs as MuSC-specific deletion of *Rptor*, an indispensable component of the mTORC1 complex, similarly impairs activation of MuSCs both in vivo and in culture [95]. Moreover, simultaneous deletion of *Tsc1* partially rescues the cell cycle re-entry defect of PI3K deficient MuSCs, which strongly supports the notion that mTORC1 is required for MuSC activation. FoxO transcription factors, which are inactivated upon AKT activation, function as another branch of downstream mediators of PI3K. Knockdown of FoxOs also partially rescues the cell cycle re-entry defect of the PI3K deficient MuSCs [79]. It seems different FoxO members play redundant roles in maintaining MuSC quiescence. While deletion of *FoxO3* alone did not cause a significant reduction of the quiescent MuSC pool, deletion of *FoxO1/FoxO3/FoxO4* together impaired quiescence maintenance of MuSCs and triggered spontaneous MuSC activation [54,96]. As both mTORC1 activation and FoxOs inhibition are required for MuSC cell cycle re-entry, it would be interesting to investigate whether and how mTORC1 and FoxOs signal with each other. Additionally, it has been shown that constitutive activation of Notch signaling prevents depletion of *Pten*-null MuSCs [33]. Consistently, we found that inactivation of PI3K signaling also prevents depletion of *Rbpj*-null MuSCs in vivo (our unpublished data). These results suggest intrinsic crosstalk between these two signaling pathways. Mechanistically, the PI3K pathway likely regulates Notch signaling via FoxOs. FoxO1 was shown to physically interact with RBPJ to promote Notch target gene expression in myoblasts, while FoxO3 was shown to directly regulate Notch receptors *Notch1* and *Notch3* in MuSCs [33,96]. In other tissues such as the retina, PI3K activation also impairs Notch signaling [97]. Conversely, Notch signaling was also found to inhibit PI3K signaling, probably through upregulating *Pten* transcription [33]. RBPJ was shown to directly bind to the *Pten* promoter in human cells, and it remains to be investigated whether similar mechanisms also operate in mouse cells [98,99,100]. Other signaling pathways that are implicated in the regulation of MuSC maintenance include the WNT and fibroblast growth factor (FGF) pathways [101,102]. WNT signaling increases upon muscle injury [103,104]. However, the role of WNT signaling seems to be complicated, as it has been implicated in many steps of MuSC lineage progression including proliferation, differentiation and self-renewal [105]. This is likely caused by different experimental models and approaches. Importantly, MuSC-specific deletion of APC, the negative regulator of canonical WNT signaling, leads to constitutive activation of β-catenin and impairs MuSC activation and survival [106]. However, MuSC quiescence is not disrupted by β-catenin activation. Consistently, MuSC-specific deletion of β-catenin did not adversely affect MuSC functions in vivo [104]. Thus, in MuSCs, β-catenin-dependent canonical WNT signaling is not required for quiescence maintenance or MuSC activation but could impede MuSC activation when constitutively activated [104,106]. Among different WNT ligands, WNT1, WNT3a and WNT5a were shown to promote MuSC proliferation while WNT4 and WNT6 were found to promote MuSC quiescence [105]. Interestingly, myofiber derived WNT4 promotes MuSC quiescence by activating non-canonical RhoA-YAP signaling and restricting MuSC migration [107]. Moreover, planar cell polarity (PCP) signaling activated by WNT7a promotes symmetric expansion of satellite stem cells [108,109]. As to the roles of FGF signaling in MuSCs, MuSCs express high levels of FGF receptors (FGFR1 and FGFR4), which, when engaged by ligands, could activate downstream signaling pathways including the ERK, p38 and PI3K pathways and play important roles in MuSC lineage progression [102]. FGFR inhibition impaired the label retention ability of MuSCs [110]. SPRY1 is a negative regulator of receptor tyrosine kinases and highly expressed in quiescent MuSCs. It is downregulated when MuSCs undergo activation and proliferation [111]. In adult mice, SPRY1 is required for the self-renewal of MuSCs after injury but not for quiescence maintenance. However, in aged mice, loss of *Spry1* leads to disruption of MuSC quiescence and accelerated cycling. The different requirements of SPRY1 for quiescence maintenance under adult and aging conditions are due to enhanced FGF2 secretion by myofibers in aged mice. Consistently, inhibition of FGFR1 activity ameliorated the depletion of MuSC with aging [51,110,111]. MuSC quiescence maintenance is also contributed by the CALCR-cAMP-PKA pathway. Loss of *Calcr* in adult MuSCs resulted in MuSC apoptosis [112]. CREB was shown to prime quiescent MuSCs for subsequent cell cycle re-entry through a CREB-MPP7-AMOT-YAP1 axis [113]. In addition, RET signaling also contributes to MuSC quiescence and self-renewal. GAS1 is a negative regulator of RET signaling and MuSC quiescence and its activity is antagonized by glial cell line-derived neurotrophic factor (GDNF), the ligand for RET [114].

### 4.2. Post-Transcriptional Regulation

Post-transcriptional regulation recently emerged as a key player in maintaining MuSCs in a poised state ready for rapid activation (Figure 1). AUF1 is an RNA binding protein promoting degradation of ARE containing mRNA. Overexpression of multiple ARE-containing transcripts due to loss of *Auf1* impaired quiescence maintenance [115]. Both *Myod1* and *Myf5* are transcribed in quiescent MuSCs, yet their proteins are only translated upon activation. Suppression of *Myod1* translation under quiescence is mediated by RNA binding proteins TTP and Staufen1, which promote degradation and translation inhibition of *Myod1* mRNA, respectively [116,117]. In addition, microRNAs are involved in the repression of *Myf5* translation under quiescence. miR-31, a microRNA highly enriched in quiescent MuSCs, sequesters *Myf5* transcripts in messenger ribonucleoprotein (mRNP) granules that are translationally inhibitory but disassociated upon activation [118]. The importance of the microRNA pathway in quiescence maintenance is further evidenced by the spontaneous activation of *Dicer*-null MuSCs [34]. miR-489, another microRNA preferentially expressed in quiescent MuSCs, contributes to quiescence maintenance by inhibiting DEK expression [34]. DEK is a splicing factor whose activity is induced upon MuSC activation. A recent study by Yue et al. identified intron retention in the transcriptome as a key mechanism employed by multiple somatic stem cells for quiescence maintenance [119]. Using in situ fixation, they found more than 1000 genes are incompletely spliced in quiescent MuSCs, while about only 50 intron retention events are detected in activated MuSCs. Partial mRNA processing prevents undesired protein translation under quiescence but ensures rapid response upon activation. Notably, *Myod1* displays strong intron retention only in the quiescent state, which provides another explanation for the absence of its protein under quiescence. Overexpression of the splicing factor DEK reduced intron retention and impaired quiescence maintenance [119]. In addition, *Pax3* is also post-transcriptionally regulated. Compared to PAX3-expressing MuSCs in the diaphragm, hindlimb MuSCs barely express PAX3 protein and have a slower activation kinetics, reflecting a deeper quiescent state. Differential expression of PAX3 in MuSCs residing in different muscles is achieved through U1 nucleolar RNA-mediated alternative mRNA polyadenylation, which generates *Pax3* transcripts with different lengths of 3′untranslated region and different susceptibilities to the regulation by miR-206 [55]. Another hallmark of quiescent stem cells is their reduced protein translation. In quiescent MuSCs, phosphorylation of translation initiation factor eIF2α at Ser51 serves as a general mechanism in restraining global translational levels [120]. Interestingly, the translation of a small subset of upstream open reading frames (uORF)-containing mRNAs is phospho-eIF2α-dependent, and the functions of these genes are related to stemness. MuSCs deficient in eIF2α phosphorylation spontaneously activate but fail to self-renew [120]. Moreover, the maintenance of proteostasis is also required for the balance between MuSC quiescence and activation. Notchless, a conserved regulator of ribosome biogenesis, is not required for MuSC quiescence maintenance. However, loss of Notchless caused severe growth defect during quiescence exit and impaired activation, highlighting the importance of differential modulation of proteostasis in MuSC quiescence maintenance and exit [121]. Consistently, Rpt3, an essential subunit of the 26S proteosome, is also required for MuSC quiescence maintenance as its deletion leads to a drastic depletion of quiescent MuSC pool due to severe apoptosis [122].

### 4.3. Epigenetic Regulation

Epigenetic mechanisms critically determine the balance between quiescence maintenance and exit (Figure 1). Compared to activated MuSCs, the chromatin of quiescent MuSCs is largely permissive, and it becomes more repressed due to acquisition of more H3K27me3 upon activation [123]. H3K27me3 levels are low in quiescent MuSCs but significantly upregulated in activated MuSCs, and many genes enriched in quiescent MuSCs, but rapidly downregulated upon activation, gain H3K27me3 at their transcription start sites (TSS) [123]. Consistently, depletion of *Ezh2*, the enzymatic core of the PRC2 complex, impaired MuSC proliferation and muscle regeneration [124,125]. Interestingly, UTX (KDM6A), an X-chromosome encoded H3K27me3 demethylase, is also required for *Myog* gene expression during MuSC lineage progression [126]. Loss of UTX in adult MuSCs also impairs their regenerative functions in vivo. H3K4me3, a histone mark for transcriptional activation, is prevalent under quiescence and similarly deposited in both quiescent and activated MuSCs. Interestingly, cell cycle-related genes are already marked by H3K4me3 in quiescent MuSCs despite the fact they are not yet transcribed [123]. Moreover, H3K4me3 deposition seems to boost the rapid induction of immediate early genes (IEGs) such as *Fosl1* and *Egr3* during early activation of MuSCs, while reduction in H3K27ac, but not H3K4me3, is involved in the downregulation of *Pax7* and *Hes1* in the same process [38]. In addition, PRC1-mediated H2AK119ub is involved in the repression of *Cdkn2a* and prevention of senescence in MuSCs [49]. Quiescent MuSCs are also known to possess high levels of facultative heterochromatin that is contributed by the H4K20 dimethyltransferase Suv4-20h1 [127]. Depletion of Suv4-20h1 reduced facultative heterochromatin formation, resulting in the transcriptional activation of *Myod1* and impairment of quiescence maintenance [127]. SIRT1, a class III HDAC, contributes to quiescence maintenance via restraining H4K16ac on myogenic genes, thus preventing precocious differentiation of MuSCs. The activity of SIRT1 requires NAD^+^, which is regulated by cellular metabolic status [128]. Indeed, it has been shown that metabolism and epigenetics are two tightly coupled cellular processes as both substrate availability and enzymatic activity required for epigenetic modifications are controlled by metabolism [129,130]. H3R8me2s, an arginine methylation contributed by PRMT5, is required to promote MuSC proliferation by suppressing *Cdkn1a* expression. Loss of *Prmt5* impaired MuSC expansion and muscle regeneration [131]. DNA methylation, another form of epigenetic modification, regulates MuSC proliferation through inhibition of *Cdkn1c* expression [132]. Moreover, HIRA, a histone chaperone, was recently shown to retain MuSC identity and to regulate self-renewal by promoting H3.3 deposition and enhancing H3K27ac levels [133]. 

### 4.4. Niche Signals

MuSCs reside in a specialized niche that is required for their maintenance, and MuSCs in turn actively remodel their niche environments to meet their own needs. Major players in the MuSC niche include other cell types such as immune cells, fibroadipogenic progenitors (FAPs) and myofibers, together with extracellular matrix and blood vessels [48,134]. The MuSC niche provides various growth factors and other ligands to regulate the balance between MuSC quiescence and activation [63,76,77,78,107,108,135,136,137,138,139] (Table 1). Disruption of the MuSC niche following injury triggers their entry into the cell cycle [3,134].

### 4.5. Metabolic Reprogramming during MuSC Quiescence Exit

MuSCs undergo extensive metabolic reprogramming upon activation, which serves as a prerequisite for cell cycle re-entry [128,140]. Quiescent MuSCs express high levels of transcripts encoding enzymes involved in FAO, suggesting their reliance on FAO for energy supply [38,128]. Indeed, inhibition of FAO in MuSCs promotes precocious differentiation and impairs muscle regeneration [141]. Preferential usage of FAO in quiescent MuSCs also coordinates the establishment of a proper epigenetic landscape by regulating the cellular NAD^+^/NADH ratio and the histone deacetylase activity of SIRT1 [128]. Consistent with the role of NAD^+^ in stem cells, NAD^+^ repletion improved stemness of aged MuSCs, prevented senescence, and enhanced their regenerative capacities [142]. Another energy source used by quiescent MuSCs is autophagy, which is further boosted upon MuSC activation to meet the biosynthetic requirement by providing nutrients such as pyruvate [49,143]. Unlike FAO-related transcripts, glycolysis-related transcripts are low in quiescent MuSCs but rapidly induced upon MuSC activation [128,144]. Although inefficient in producing ATP compared to OXPHOS, glycolysis provides essential metabolic intermediates for cell growth and biosynthesis that are, in turn, required for cell cycle re-entry. Glycolytic intermediates directly feed the production of nucleotides and amino acids as well as lipid molecules. The induction of glycolysis upon MuSC activation resembles the Warburg effect observed in cancer cells [145]. Transcription factor YY1 also plays a role in coordinating glycolysis and mitochondrial respiration during MuSC early activation [140]. Although required for MuSC early activation, glycolysis seems to be dispensable in proliferating myoblasts, where glucose is mainly used to maintain histone acetylation levels by providing acetyl-CoA [146]. Compared to activated MuSCs, both mitochondria numbers and activities are low in quiescent MuSCs [62,144,147]. Upregulation of OXPHOS upon MuSC activation also elicits a burst in ROS levels [147], the dysregulation of which could trigger cellular senescence or apoptosis [144,147,148]. Of note, blocking either glycolysis or electron transport chain activity in MuSCs strongly prevents quiescence exit and the subsequent cell cycle re-entry, highlighting the importance of metabolic reprogramming during early MuSC activation [140,144]. Indeed, such metabolic reprogramming is conserved among multiple quiescent tissue-resident stem cells and progenitor cells during their activation, although the exact roles of individual metabolic pathways might be context-dependent [4,36,48,149]. For example, induction of glycolysis is also required for the early activation of naïve T cells and HFSCs, whereas it plays a more important role in quiescence maintenance in HSCs and NSCs [65,150,151,152,153,154,155,156]. However, such views are challenged by a recent study demonstrating that glycolysis is not the main energy source for quiescent HSCs [157]. Similarly, in multiple adult quiescent stem cells, including HSCs, NSCs and quiescent progenitor cells such as naïve T cells, enhanced mitochondrial biogenesis and respiration are crucial for their activation, while FAO plays a more important role in quiescence maintenance [149,151,155,158,159,160]. Such utilization of similar metabolic pathways in different adult stem cells reinforces the importance of metabolic reprogramming as an important regulatory mechanism during stem cell activation.

## 5. Multiple Checkpoints during MuSC Quiescence Exit

**Figure 2:**
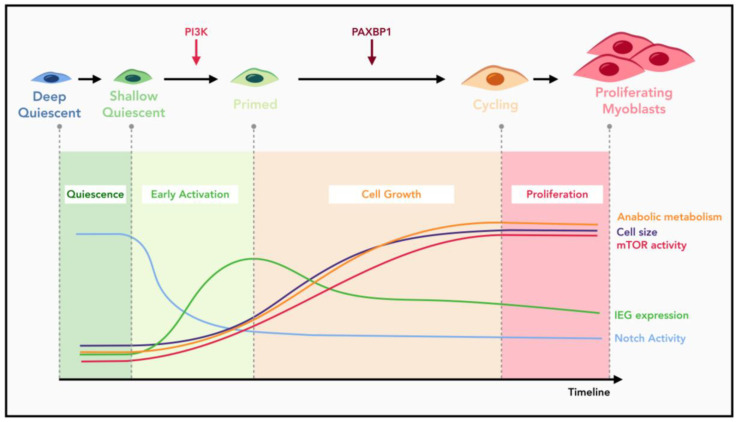
Schematic showing the progression of MuSC quiescence exit. Quiescent MuSCs are a pool of heterogeneous stem cells with different depths of quiescence. The more dormant MuSC population has slower activation kinetics but typically possesses stronger engraftment capacities. Early activation represents the first stage of quiescence exit, which happens within hours post tissue disruption. Key features of this stage are rapid induction of PI3K signaling and induction of many IEGs. This is followed by a long cell growth stage fueled by continuously increasing mTORC1 activity and coordinated metabolic reprogramming. PI3K and PAXBP1 are two critical regulators during quiescence exit that control an early activation checkpoint and a cell growth checkpoint, respectively.

MuSC quiescence exit describes the transition from quiescence into S phase of the first cell cycle, a process that takes ~36 h and is about four times longer than that for subsequent cell cycles [16,62,66,68,144]. The complexity of this transition is further evidenced by the recently identified early activation process, a short time window during which MuSCs undergo dramatic epigenetic and transcriptomic changes [38,161]. Through in situ fixation and in vivo labeling of nascent transcripts, it is now possible to capture the dormant MuSC state and pinpoint the changes happening during early activation process. Key alterations during this stage include upregulation of genes involved in ribosomal biogenesis and downregulation of genes involved in FAO [38,119]. Our laboratory previously demonstrated that this early activation process is critically controlled by the PI3K-mTORC1-FoxO axis [79]. Loss of p110α of PI3K arrested MuSCs in the quiescent stage, which can be partially rescued through deliberate activation of mTORC1 via simultaneous inactivation of the TSC complex. FoxOs are also involved in the quiescence maintenance of MuSCs, as knockdown of FoxOs also partially rescued the cell cycle re-entry defect of p110α-null MuSCs, which is consistent with the recent FoxO genetic knockout study [54]. Therefore, PI3K functions to regulate the earliest known checkpoint during MuSC activation to determine whether MuSCs can initiate the process of quiescence exit [79] (Figure 2). As to the G_alert_ state, it was initially identified in MuSCs from the uninjured muscles contralateral to the injured ones [62]. MuSCs in the G_alert_ stage are slightly larger in size, with enhanced mitochondria activity and transcriptional levels. However, such increases are still much lower compared to those seen in fully activated MuSCs. Most importantly, entry into the G_alert_ stage is reversible and cells can return to the more dormant or deeper quiescent stage from the G_alert_ state [4,62], making MuSCs in the G_alert_ stage different from those passing the PI3K-controlled checkpoint. Therefore, the extent to which PI3K signaling is activated, and the persistence of the activated signaling, might determine the fate choice of quiescent MuSCs. While constitutive activation of PI3K by either *Pten* deletion or expression of an oncogenic form of p110α is sufficient to drive spontaneous quiescence exit and cell cycle re-entry of MuSCs, genetic activation of mTORC1 by *Tsc1* deletion only allows MuSCs to enter the reversible G_alert_ stage [33,62,79]. Interestingly, inactivation of *Tsc1* in HSCs is sufficient to promote their full activation and cell cycle entry [162]. It is likely that simultaneous inactivation of FoxOs is also required in the context of MuSC activation [79].

After passing the initial PI3K-dependent checkpoint, MuSCs still need to undergo substantial cell growth concomitant with enhanced mTORC1 activity before their re-entry into the first cell cycle (Figure 2). Recently, we identified a second activation checkpoint during MuSC quiescence exit that is controlled by PAXBP1 [144,163]. *Paxbp1*-null MuSCs were able to pass the PI3K-dependent checkpoint as evidenced by the initial upregulation of mTORC1 activity, an increase in cell size, expression of MyoD protein, and induction of many IEGs (Figure 2). However, *Paxbp1*-null MuSCs were arrested at a later stage during cell growth with concomitant failures in further upregulation of mTORC1 signaling [144]. As a result, mutant MuSCs were blocked from cell cycle re-entry and underwent apoptosis. Mechanistically, PAXBP1 is required for optimal activation of mTORC1 signaling by controlling cellular ROS levels. It remains to be explored whether other important activation checkpoints exist during prolonged MuSC quiescence exit.

## 6. A Conserved Role of mTORC1 Signaling in Quiescence Exit

mTORC1 activity is low in quiescent MuSCs, but is rapidly induced upon early activation [62,79]. Moreover, activation of mTORC1 also drives the entry of MuSCs into the G_alert_ stage. The initial activation of mTORC1 is totally dependent on the PI3K activity, as depletion of p110α of PI3K or *Rptor* severely blocked MuSC activation and muscle regeneration [79,95]. On the contrary, forced activation of mTORC1 through ablation of *Pten* or using a constitutive active form of p110α triggers spontaneous activation of MuSCs [33,79]. Thus, mTORC1 activity functions as a key molecular switch: it is off in quiescent MuSCs but rapidly turned on upon MuSC activation. Moreover, our group demonstrated that there is a continuous increase in mTORC1 activity throughout the entire quiescence exit process [144] (Figure 2). While p110α of PI3K is required to trigger the initial mTORC1 activation, the subsequent enhancement of mTORC1 activity is dependent on PAXBP1 and induction of many PAXBP1-controlled antioxidant genes [144]. In the absence of PAXBP1, the first wave of mTORC1 activation is unaffected. However, the subsequent increase in mTORC1 activity is totally abolished, resulting in impaired cell growth and metabolic reprogramming. As a result, the mutant MuSCs fail to re-enter the cell cycle and undergo a p53-dependent apoptosis. Blocking the second wave of mTORC1 activation with chemical inhibitors similarly prevents MuSC from cell cycle re-entry [144].

As a master regulator of cellular metabolism and cell growth, the essential roles of mTORC1 signaling in quiescence exit is conserved among multiple adult stem cells [164,165]. Quiescence maintenance of HSCs requires suppression of mTORC1 activity by miRNAs expressed from the *Dlk1-Gtl2* locus [166]. Deliberate activation of mTORC1 signaling triggers quiescence exit and depletion of HSCs [32,162,167]. The expansion of quiescent NSCs in the mouse forebrain is also dependent on the activation of mTORC1 [168]. Similarly, mTORC1 is activated during the transition from telogen to anagen in HFSCs and inhibition of mTORC1 activity delayed HFSC activation and anagen initiation [169]. In addition, mTORC1 is also required to orchestrate reprogramming of major metabolic pathways including glycolysis, lipid biosynthesis and mitochondrial respiration during activation of quiescent native T cells [156,170,171]. Loss of mTORC1 activity abolished their exit from quiescence. Thus, mTORC1 is indispensable during activation of multiple types of quiescent cells including both adult stem cells and other tissue progenitor cells.

## 7. Conclusions and Future Directions

Proper muscle functions are essential for good quality of life, and manipulation of satellite cells are promising strategies to ameliorate the impaired muscle functions during aging and pathological conditions. Much progress has been made to understand the behaviors of MuSCs during muscle regeneration, yet to better facilitate utilization of MuSCs for muscle therapies, many fundamental questions regarding intrinsic MuSC biology and their interaction with the niche remain to be solved. In vitro cultured MuSCs suffer from loss of stemness following expansion, and different regimens have been proposed to ameliorate this limitation [45,120,172]. A better understanding of the regulatory mechanisms underlying stem cell quiescence maintenance, quiescence exit, myoblast proliferation and differentiation, and self-renewal, will eventually lead to more efficient and effective MuSC-based regenerative therapies.

Previous methods for adult stem cell isolation required extensive tissue dissection and digestions, which were recently shown to elicit stress responses in stem cells. As a result, the transcriptome profiles of freshly isolated stem cells were skewed by disassociation-induced artefacts and may have failed to represent the genuine quiescent state [173,174,175]. Thus, previous transcriptome profiles derived from freshly isolated MuSCs should be revisited. New methods aiming to preserve the true quiescent state of stem cells by employing in situ fixation or nascent mRNA tagging have already advanced our understanding of the true quiescence signatures [38,119,161,174]. For example, IEGs such as *Jun* and *Fos* are highly expressed in freshly isolated MuSCs, but their mRNA levels are actually much lower in fixed MuSCs [38,175]. In situ fixation also revealed a prevalent intron-retention mechanism in the transcriptomes of quiescent stem cells, which was previously overlooked due to technical limitations [119]. While in situ fixation followed by scRNA-seq or single nucleus RNA-seq (snRNA-seq) is powerful for unbiased identification of genuine quiescent stem cells, this suffers from the loss of important spatial information and therefore has limited applications in deciphering cellular crosstalk within the stem cell niche.

Among cutting-edge technologies, spatial transcriptomics/genomics based on either sequencing or imaging platforms provide greater opportunities to capture MuSCs in true quiescent state to characterize MuSC heterogeneity and their niche diversity, as well as to dissect the regulatory mechanisms of quiescence maintenance and exit. Sequencing-based spatial transcriptomic profiling with Slide-seq or sci-Space that resolve spatial heterogeneity at or near single cell resolution are ideal tools to study the dynamics of MuSC niche and cellular crosstalk during muscle regeneration [176,177]. Moreover, integrated multimodal approaches from DNA seqFISH+ may allow comprehensive comparison of genome architecture, epigenetic modifications, and subcellular bodies, as well as gene expressions between different MuSC pools such as satellite stem cells and activated satellite cells on the same tissue sections [15,178,179].

Moreover, molecular profiling at the protein level is essential to understand the post-transcriptional and post-translational regulation of MuSC quiescence maintenance and exit. Single cell technologies such as Cite-seq and INs-seq now allow simultaneous measurements of protein expression or activity together with traditional transcriptomic profiles [180,181]. Proteomic analysis at the single cell level, such as cytometry by time of flight (CyToF), will continue to broaden our understanding of the dynamics of MuSCs and their niche during physiological and pathological conditions [56,61]. Particularly, newly developed technical platforms, such as Codex, now enable visualization of dozens of proteins on tissue sections, which holds great promise in dissecting the dynamics of MuSC niche and immune cell infiltration during muscle regeneration. Overall, a better understanding of essential intrinsic and extrinsic regulatory mechanisms underlying MuSC quiescence maintenance and exit will undoubtedly promote the development of efficient and effective MuSC-based regenerative therapies and alleviate various muscle disorders.

## Figures and Tables

**Table 1:** Known niche factors regulating MuSC quiescence and activation.

Niche Factors	Sources	Functions	References
OSM	Muscle fibers	Induce MuSC quiescence	Sampath et al., 2018 [135]
WNT4	Muscle fibers	Maintain MuSC tension and quiescence	Eliazer et al., 2019 [107]
HGFA	Serum	Process pro-HGF and promote MuSC entry into G_alert_ state	Rodgers et al., 2017 [63]
COLV	MuSC	Interact with Calcitonin receptor and maintain MuSC quiescence	Baghdadi et al., 2018 [75,76]
Fibronectin	MuSC	Stimulate WNT7A signaling and MuSC expansion	Bentzinger et al., 2013 [108]
FGF2	Muscle fibers	Expression of FGF2 in aged muscle fibers breaks MuSC quiescence	Chakkalakal et al., 2012 [51]
COLVI	Fibroblasts	Required for MuSC self-renewal after injury	Urciuolo et al., 2013 [136]
DLL4	Newly formed myotubes/endothelial cells	Activate Notch signaling and promote MuSC quiescence	Low et al., 2018 [77]; Verma et al., 2018 [78]
NCAD/MCAD	Muscle fibers	Forms adhesive junctions between MuSC and myofibers and maintain MuSC quiescence	Goel et al., 2017 [137]
ADAMTS1	Macrophages	Reduce Notch signaling and induce MuSC activation	Du et al., 2017 [138]
WISP1	FAP	WISP1 stimulates asymmetric MuSC commitment	Lukjanenko et al., 2018 [139]

## Data Availability

Not applicable.
